# Electroconvulsive Therapy in Schizophrenia: Exploratory Study on the Efficacy, Cognitive Effects, and Safety of Right Unilateral Versus Bitemporal Techniques

**DOI:** 10.1002/brb3.71351

**Published:** 2026-03-26

**Authors:** Héctor O. Castañeda‐González, Alexandra Magaña‐Ornelas, Aurea Ruby Santiago‐Maury, Sophia Sánchez López, Iván de Jesús Leonor‐Hernández, Hector Cabello‐Rangel

**Affiliations:** ^1^ Fray Bernardino Álvarez Psychiatric Hospital Mexico City Mexico

**Keywords:** cognitive funtions, efficacy, electroconvulsive therapy, schizophrenia

## Abstract

**Introduction:**

Electroconvulsive therapy (ECT) is an effective treatment for resistant schizophrenia, although debate persists about the optimal electrode placement technique. Bitemporal stimulation (BTS) is the most commonly used, while right unilateral stimulation (RUS) is associated with fewer cognitive effects in other disorders.

**Methods:**

Exploratory study including 17 patients randomized to BTS (*n* = 8) or RUS (*n* = 9). Symptoms and severity were assessed with the Positive and Negative Symptom Scale (PANSS) and Clinical Global Impression (CGI) scale, cognitive functions with Montreal Cognitive Assessment (MoCA) and Brief Assessment of Cognitive in Schizophrenia (BACS). Symptomatic changes, frequency and time of onset of side effects, changes in cognitive performance, and specific functions were compared.

**Results:**

Both groups showed significant improvement in symptoms (ΔPANSS: BTS = 44.75 vs. RUS = 39.11; *p* = 0.724), with no differences in response rates (75% BTS vs. 44.4% RUS; *p* = 0.335). RUS required a lower stimulus to induce seizures (29.8 mC vs. 54 mC in BTS; *p* = 0.003). The BTS group showed deterioration in verbal fluency (BACS: *p* = 0.042), while the RUS showed improvement in motor speed (*p* = 0.046). There were no global differences in MoCA or BACS.

**Conclusion:**

Both techniques are equally effective in symptomatic reduction, but RUS could offer advantages as it requires less load and is associated with less deterioration in verbal fluency. These preliminary results have methodological limitations, mainly the sample size and lack of power calculation, but they may encourage further research.

**Trial Registration:**

Clinicaltrials.gov identifier: NCT06972745

## Graphical abstract

The purpose of the study was to compare the effectiveness, safety, and cognitive effects of BTS versus RUS in hospitalized patients with schizophrenia. The results should be considered preliminary due to the sample size (*n* = 17) and lack of blinding of participants.

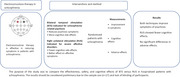

## 1 Introduction

Electroconvulsive therapy (ECT) is an effective treatment for people with schizophrenia (Ali et al. 2019; Chan et al. 2019). Schizophrenia treatment guidelines suggest its use in treatment resistance and some clinical risk situations (e.g., suicidal risk). However, its usefulness has been described throughout the natural history of the disease (Grover et al. 2019). The discussion about the optimal modality in the placement of stimulation electrodes is old (Kellner et al. 2010). Bitemporal stimulation (BTS) is the most widespread technique due to the limited evidence supporting the use of right unilateral stimulation (RUS) (Grover et al. 2019). RUS is recommended for affective disorders in the context of bipolar disorder or treatment‐resistant depression, as it has shown fewer cognitive side effects (Abbott et al. 2019). There is still a gap in knowledge about the efficacy of RUS placement in schizophrenia due to the methodological limitations of previous studies (Abraham and Kulhara 1987; Doongaji et al. 1973; el‐Islam et al. 1970; Wessels 1972). The aim of this study was to compare the efficacy and safety of BTS versus RUS in patients with schizophrenia. The specific objectives were to (a) compare the variation in scores on the Montreal Cognitive Assessment (MOCA) and the Brief Assessment of Cognitive in Schizophrenia (BACS) and (b) compare the variation in improvement with Clinical Global Impression Scale.

## 2 Methods

### 2.1 Study Design and Setting

Exploratory study with parallel random assignment BTS conventional intervention or RUS was the intervention to be compared. Patients were randomly assigned to one or the other intervention by a blinded investigator.

### 2.2 Sample Size

**FIGURE 1:**
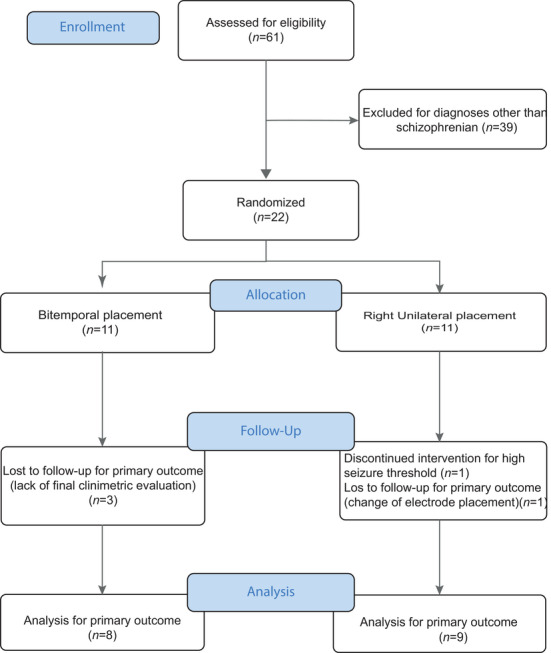
Flowchart of patients included in the study.

Sixty‐one patients were eligible over the course of 1 year, but 39 were excluded. The remaining 22 were randomized to one intervention or the other. A list of random numbers was generated in Excel for randomization (Figure 1).

### 2.3 Eligibility Criteria

The eligibility criteria were as follows: Spanish‐speaking patients of any sex or gender; at least 18 years of age, with a diagnosis of schizophrenia according to criteria of the Diagnostic and Statistical Manual of Mental Disorders in its fifth edition (DSM‐5); and score ≥ 60 on the Positive and Negative Symptom Scale (PANSS) to ensure moderate symptom severity for ECT indication (Leucht et al. 2005). patients with antipsychotic treatment were included (clozapine were accepted).

Patients who received ECT in the last 3 months, with affective comorbidities, catatonic syndrome, pregnancy, and contraindications to general anesthesia or ECT were excluded. Patients who did not complete the final evaluation or treatment were eliminated.

### 2.4 Equipment and Technique

A spECTrum 5000Q equipment from MECTA (USA) was used and certified and calibrated annually according to the manufacturer's recommendations. The lead author, who applied the treatment, is certified by Emory University (GA, USA).

According to the psychiatric hospital's ECT protocol, the patient undergoes electrocardiogram studies, plain chest x‐ray, blood count, blood chemistry, and serum electrolytes. An internist and an anesthesiologist determine the cardiovascular risk.

ECT is performed in a space designated for this purpose. An anesthesiologist is responsible for sedation and muscle relaxation, using atropine (1 mg, intramuscular), propofol 2 mg/kg intravenous, and succinylcholine 1 mg/kg intravenous. The total number of sessions was decided by the treating physician. Each patient receives three ECT sessions per week, resting Saturday and Sunday. The number of sessions and the end of treatment was decided by the treating physician according to the clinical evolution.

We use frontotemporal placement for BTS as the standard position for schizophrenia‐related psychosis (Grover et al. 2019). Right temporoparietal placement is performed according to the D'Elia method; this position is more suitable for affective disorders as it has fewer cognitive effects (Malhi et al. 2021).

For threshold titration, loading was started at 48 mC (0.5 ms/20 Hz/1.5s/0.8A) for patients with BTS and 4.8 mC (0.3 ms/20 Hz/0.5s/0.8A) for RUS, according to Emory University ECT Titration Schedule. If the seizure threshold was not reached, the dose was doubled until an adequate quality seizure was induced.

The quality of the crisis was assessed using the CASBAS method (Brunner and Grözinger 2018). The subsequent therapeutic loads were 2x the threshold for BTS and 6x for RUS, adjusting by 50% in case of inducing a poor‐quality crisis.

The PANSS and Clinical Global Impression (CGI) scales were applied prior to treatment and 48 h after the last ECT session. The MoCA instrument is validated in the Mexican population. The BACS scale was used to evaluate the cognitive domains verbal memory, working memory, motor speed, verbal fluency, attention, executive functions, validated in the Mexican population. It was decided to apply both cognitive assessments to compare their usefulness in detecting cognitive changes secondary to ECT. The MoCA and BACS scales were applied at two times, 24 h prior to the first ECT session and 48 h after the last session. Three researchers were trained in the application of all the tests by a specialist in neuropsychology and psychopathology.

### 2.5 Intervention and Comparator

The study groups were a RUS group (intervention) and conventional intervention was the BTS group. A control group (placebo) was not used due to ethical considerations, as the patients had severe psychosis.

### 2.6 Adverse Effects

Screening for side effects was performed after each ECT session, throughout the cycle of ECT sessions. The most frequent side effects measured were headache, myalgia, nausea, dizziness, episodic memory failures, or autobiographical memory.

### 2.7 Randomization, Blinding, and Allocation Concealment

Randomization (BTS or RUS) was performed using a list generated in Microsoft Excel by a blinded researcher. The assignment to each group was performed each time a patient was recruited. The result of randomization was communicated to the principal investigator before treatment was initiated.

### 2.8 Data Analysis

Sociodemographic and clinical characteristics were described. Continuous variables with normal distribution were presented as mean ± standard deviation (SD), continuous variables with asymmetric distribution as median/interquartile range (IQR), and categorical variables as frequency and percentage (%).

The effect size for the PANSS was calculated using the following formula: Δ PANSS = [(Pt2 − *N*) − (Pt1 − *N*)/(Pt1 − *N*)] × 100; where Pt1 = initial score, Pt2 = final score, and *N* = total score (Leucht et al. 2007). A reduction of ≥ 30% from baseline and an improvement of 1 point on the CGI scale was considered therapeutic efficacy (responder/nonresponder) (Leucht et al. 2005; Samara et al. 2019).

The Student's *t*‐test for paired samples was used to compare the intragroup baseline and final scores. Additionally, the Student's *t*‐test for unpaired samples was used to compare the intergroup effect size.

The efficacy of the treatment was compared using Fisher's exact test. *Safety assessment*: The frequency and severity of post‐ECT adverse effects were compared, comparing both the time of onset and distribution using survival models and the *χ* ^2^ test. *Cognitive effects*: Neurocognitive impact was assessed by calculating the Δ in MoCA and BACS (final − baseline). The Student *t*‐test for related samples was used for intragroup comparison, while intergroup differences were analyzed with nonparametric statistics due to the free distribution of variables.

## 3 Results

58% of the sample were men, with a mean age of 37.1 years (range: 21–59 years). Regarding educational level, 35.3% of the patients had basic education, 35.3% had upper secondary education, 17.6% had postgraduate studies, 5.9% had undergraduate studies, and 5.9% only knew how to read and write.

The 58% of patients received clozapine mean dose = 318 mg/day. Among them, 31% were treated with two antipsychotics. Only three patients (15%) had a clinical history of ECT. When comparing the groups, there were no statistically significant differences in sociodemographic or clinical variables (Table 1).

**TABLE 1:** | Sociodemographic and clinical variables by ECT stimulation group.

Variable	Bitemporal (*n* = 8)	Unilateral right (*n* = 9)	*p* value
Sex			
Women (*n*)	44.4% (4)	30% (3)	0.637^a^
Men (*n*)	55.6% (5)	70% (7)	
mean age ± SD	36.1 ± 15.6	38.8 ± 9.9	0.664^b^
Schooling			
Basic (*n*)	25.0% (2)	44% (4)	0.620^a^
Upper middle school (*n*)	50% (4)	22.2% (2)	0.335^a^
Postgraduate (*n*)	12.5% (1)	22.2% (2)	1.0^a^
Duration of illness			
< 5 years	37.5% (3)	22.2% (2)	0.620^a^
5–10 years old	25% (2)	22.2% (2)	1.0^a^
> 15 years	37.5% (3)	22.2% (2)	0.620th
History of ECT	25% (2)	11.1% (1)	0.576^a^
Treatment with clozapine	75.0% (6)	55.5% (5)	0.620^a^
mean dose of clozapine mg/day ± SD	265 ± 174.2	168 ± 183.1	0.254^b^
Patients on two antipsychotics	37.5% (3)	22.2% (2)	0.620^a^
Treatment‐resistant patients	25% (2)	44.4% (4)	0.620^a^

^a^Fisher's exact test.

^b^Mann–Whitney *U* test.

The most frequent comorbidities were treated hypothyroidism (23.5%), followed by overweight (11.7%), treated dyslipidemia (11.7%), and cannabis use (11.7%). One case of tobacco use disorder (5.8%) and another of alcohol use disorder.

The Mann–Whitney *U* test demonstrated significant differences in seizure threshold (54 mC in BTS vs. 29.8 mC in RUS, *p* = 0.003), with the same test no differences were observed in the average number of sessions per cycle (7.25 vs. 8, *p* = 0.324), nor in the maximum load administered in each group (323.25 mC vs. 350.40 mC, *p* = 0.735).

### 3.1 Response to Treatment

The PANSS variable met the assumption of normality in both groups (Shapiro–Wilk: BT *W* = 0.971, *p* = 0.913; (RUS *W* = 0.975, *p* = 0.769), and the homogeneity of variances was verified by Levene's test (*F* [1,15] = 4.218, *p* = 0.058).

Both groups showed significant symptomatic improvement at the end of treatment, with a large effect size (*t* = 5.667, 95% CI: 26.07–63.42, *p* < 0.001, *g* = 1.894 for BTS and *t* = 3.018, 95% CI: 9.23–68.99, *p* = 0.017, *g* = 0.958 for the RUS group). When comparing the groups, none showed superiority in symptomatic improvement (*W* = 44.75 in BTS vs. *W* = 39.11 in RUS, *t* = 0.360 95% CI: −27.74 to 39.02, *p* = 0.724).

At the end of treatment, 10 patients (58.8%) met the definition of response (reduction ≥ 30% PANSS + improvement in CGI), the response rate showed no significant differences in Fisher's exact test (75% in BTS vs. 44.4% in RUS, *p* = 0.335).

### 3.2 Side Effects

No serious side effects were reported, most side effects occurred after the third session (mean = 3, SD = 1.58, CI: 0–6). In the risk analysis, we found no significant difference in the time of onset of side effects between the groups (*χ* ^2^ = 0.190, *p* = 0.663, with the log rank test).

In the RUS group, two cases of arterial hypertension and one bradycardia event were documented, while in the BTS group one case of hypotension was recorded. Comparative analysis of common symptoms revealed no significant differences in the incidence of. headache (*χ* ^2^ = 0.018, *p* = 0.893) and tachycardia (*χ* ^2^ = 0.142, *p* = 0.707) using Pearson's chi‐square tests

### 3.3 Cognitive Effects of ECT

For the analysis of cognitive changes, the sample consisted of nine patients in the BTS group and seven in the RUS group.

The variables ΔMoCA (BTS: *W* = 0.866, *p* = 0.11; RUS: *W* = 0.922, *p* = 0.482) and ΔBACS (BTS: *W* = 0.962, *p* = 0.823; RUS: *W* = 0.966, *p* = 0.872) met the assumption of normality with the Shapiro–Wilk test. The homogeneity of variances was confirmed by the Levene test for ΔMoCA (*F*[1,14] = 1.937, *p* = 0.186) and ΔBACS (*F*[1,14] = 2.854, *p* = 0.113). In intragroup comparisons, only the RUS group showed a statistically significant improvement in the total posttreatment MoCA score, with a large effect size (*g* = −2.762, paired *t*‐test). In contrast, no significant changes were detected in the overall BACS scores for any group (Table 2).

**TABLE 2:** | Cognitive changes due to electroconvulsive therapy by stimulation groups.

Test	Group	Pre‐ECT (mean ± SD)	Post‐ECT (mean ± SD)	Change (mean ± SD)	*t* (95%CI)	*p*‐value
MoCA	BTS	16.67 ± 6.10	18.56 ± 6.82	−1.88 ± 3.14	−1.805 (−4.30 to 0.525)	0.109
	RUS	16.14 ± 7.26	19.43 ± 6.77	−3.28 ± 1.11	−7.813 (−4.31 to 2.25)	< 0.001*
BACS	BTS	−0.78 ± 18.43	−5.22 ± 20.97	4.44 ± 16.28	0.819 (−8.06 to 16.95)	0.436
	RUS	−6.57 ± 26.77	−0.71 ± 21.49	−5.85 ± 9.58	−1.617 (−14.71 to 3.0)	0.157

Abbreviation: SD, standard deviation.

*t for paired events.

No statistical difference was observed between the groups in the global MoCA assessment (BTS: Δ1.89 ± 3.14 vs. RUS: Δ3.29 ± 1.11; *t* = −1.11, 95% CI [−4.08, 1.28], *p* = 0.283) or in the total BACS score (BTS: Δ−4.44 ± 16.28 vs. RUS: Δ5.86 ± 9.58; *t* = −1.48, 95% CI [−25.23, 4.62], *p* = 0.161).

### 3.4 Analysis by Cognitive Domains

In motor speed (BACS), the RUS group showed significant improvement (*W* = 1.991, *p* = 0.046), although we did not observe a difference when comparing both groups (*p* = 0.396). Conversely, the BTS group experienced a deterioration in verbal fluency in the BACS battery (*W* = −2.032, *p* = 0.042), with a significantly lower performance than the RUS group in this domain (Mann–Whitney *U*, *p* = 0.025). The rest of the changes in specific cognitive functions are presented in Table 3.

**TABLE 3:** | Changes by cognitive domain within the group and between stimulation groups.

Cognitive function	Group	Median Pre‐ECT (IQR)	Median Post‐ECT (IQR)	*W* statistic	*p*‐value	Intergroup difference	*p*‐value
Visuospatial	BTS	1.78 (1–2.5)	2.66 (1–4.5)	−1.511	0.131	0.62	0.404
	RUS	1.42 (1–2)	1.71 (0–3)	−0.742	0.457		
Attention (Digit span/concentration/subtraction)	BTS	2.55 (1–4)	3 (3–3)	−2.06	0.039*	1.18	0.435
	RUS	2.71 (0–5)	4.28 (1–6)	−1.929	0.053		
Language (repetition and phonemic fluency)	BTS	1.44 (0.5–2)	1.77 (1–2.5)	−0.828	0.408	0.37	1.0
	RUS	1 (0–2)	1.43 (1–2)	−1.133	0.256		
Abstraction	BTS	1.22 (0.5–2)	1.33 (0–2)	−0.577	0.564	0.12	0.944
	RUS	1.14 (0–2)	1.28 (0–2)	−1.00	0.317		
Short‐term memory	BTS	1.89 (0–3.5)	1.55 (0–3)	−0.680	0.496	−0.25	0.909
	RUS	2.14 (0–5)	2 (0–4)	−0.577	0.563		
Orientation	BTS	4.78 (3.5–6)	4.77 (4–6)	0	1	0.43	0.184
	RUS	4.71 (5–6)	5.71 (5–6)	−1.889	0.058		
Verbal memory	BTS	18.11 (10.5–29.0)	13.88 (5.5–21.5)	−0.474	0.634	−0.68	0.222
	RUS	13.71 (3.0–24.0)	17.57 (10.0–23.0)	−0.931	−0.931		
Working memory	BTS	18.22 (9.0–26.0)	16.44 (6.0–27.0)	−0.105	0.916	0.12	0.669
	RUS	15.85 (‐11.0–25.0)	18.42 (8.0–28.0)	−0.525	0.599		
Motor speed	BTS	6.88 (−3.0–19.0)	8.66 (−9.5–28.0)	−0.296	0.767	4.37	0.396
	RUS	10.14 (0–26.0)	17.85 (4.0–32.0)	−1.991	0.046*		
Verbal fluency (semantic and phonemic)	BTS	35.22 (22.5–42.0)	26.33 (17.0–33.0)	−2.032	0.042*	−2.12	0.025**
	RUS	26.57 (16.0–33.0)	33.14 (21.0–53.0)	−1.362	0.172		
Attention (digit encoding)	BTS	13.66 (5.0–23.5)	18.66 (13.0–25.5)	−1.264	0.205	3.12	0.631
	RUS	10.57 (−2.0–22.0)	11.29 (‐3.0–22.0)	−0.135	0.892		
Executive functions	BTS	27.33 (3.0–48.0)	18.55 (−6.0–48.0)	−1.120	0.262	−5.12	0.265
	RUS	19.57 (−15.0–41.0)	19.14 (−4.0–49.0)	−0.630	0.528		

Abbreviation: IQR, interquartile range (Q3–Q1).

*Wilcoxon test.

**Mann–Whitney *U*.

## 4 Discussion

ECT remains one of the most effective interventions for treatment‐resistant schizophrenia, yet optimal electrode placement continues to be debated. In this randomized study, both bitemporal and unilateral stimulation were associated with significant intragroup symptomatic improvement.

However, no statistically significant differences were detected intergroups in overall symptom reduction or response rates, possibly due to the sample size, so that the absence of differences between groups should not be interpreted as meaning that the interventions are equivalent.

The magnitude of symptomatic improvement observed in both groups is consistent with previous literature supporting the efficacy of ECT in schizophrenia (Sinclair et al. 2019; Tor et al. 2017). Prior comparative studies evaluating electrode placement have also reports comparable clinical outcomes across configurations, although most have been limited by small samples or naturalistic designs (Ali et al. 2019; Tor et al. 2017; Bansod et al. 2018). Our findings align with this body of evidence but should be understood as preliminary and hypothesis‐generating rather than confirmatory.

One notable finding was the lower seizure threshold observed in the RUS group. This is consistent with known biophysical proprieties of unilateral stimulation using ultrabrief pulses and may reflect differences in current distribution and impedance (Kellner 2018).

While reducer charge exposure has been hypothesized to minimize cognitive side effects, this theoretical advantage cannot be established from the present data.

The relationship between electrical dose, hippocampal enlargement, and cognitive outcomes remains complex and likely moderated by multiple clinical and neurobiological variables (Argyelan et al. 2021; Denier et al. 2023; Wilkinson et al. 2017).

Regarding cognitive outcomes, no significant intergroup differences were found in global MoCA or total BACS scores. In intragroup analyses, the BT group showed a decline in verbal fluency, whereas the ULD group demonstrated improvement in motor speed and global MoCA score. These findings should be interpreted with caution for several reasons. First, cognitive assessments were conducted 48 h after final ECT session, capturing acute posttreatment effects rather than long‐term cognitive trajectories. Second, multiple cognitive domains were analyzed without correction for multiple comparisons, increasing the possibility of type I error. Third, the small sample limits the reliability and generalizability of domain‐specific findings.

The heterogeneity of cognitive changes observed in the study is consistent with prior research in schizophrenia, where post‐ECT cognitive trajectories vary depending on baseline cognitive status, timing of assessment, and electrode placement (Vaccarino and Vaccarino 2024). Importantly, the present study was not powered to detect modest cognitive differences between stimulation modalities. Therefore, no definitive conclusions can be drawn regarding comparative cognitive safety.

In terms of safety, no serious adverse events occurred, and the frequency and timing of common side effects did not differ significantly between groups. Although the clinician‐determined duration of treatment reflects real‐world practice, the absence of standardized stopping criteria represents a potential source of variability and should be considered when interpreting results.

Several methodological limitations should be acknowledged. The sample size was small, and no a priori power analysis was performed, which limited statistical power. Analyses were performed according to protocol without imputation of missing data, which may introduce bias. Outcome assessors were not blinded to treatment assignment, which could introduce assessment bias. In addition, cognitive follow‐up was limited to the period immediately following treatment. These factors collectively limit the strength of inferences. The study was registered retrospectively.

Despite these limitations, random assignment to each group, the standardized stimulation protocol, and structured cognitive assessment represents methodological strengths. The study provides preliminary data in an area with limited evidence and highlights the need for trials with large sample sizes that incorporate long‐term cognitive follow‐up.

## 5 Conclusions

In this exploratory randomized study of hospitalized patients with schizophrenia, both bitemporal and right unilateral ECT were associated with significant intragroup symptomatic improvement. No statistically significant differences were detected between stimulation modalities in overall symptom reduction or response rates; however, the study was not powered to determine equivalence or non‐inferiority.

RUS required a lower seizure threshold, consistent with its biophysical characteristics. Short‐term cognitive findings were heterogeneous and limited to the immediate posttreatment period, and should therefore be interpreted cautiously.

Given the small sample size, lack of power calculation, absence of blinding, and short cognitive follow‐up, these findings should be considered preliminary. Larger, prospectively registered, adequately powered trials with standardized treatment duration and longer term cognitive assessment are required to clarify the comparative clinical and cognitive profiles of electrode placement strategies in schizophrenia.

## Author Contributions

**Héctor O. Castañeda‐González**: conceptualization, investigation, project administration, data curation, validation methodology, writing – review and editing, writing – original draft. **Alexandra Magaña‐Ornelas**: methodology, validation, writing – review and editing, investigation, data curation. **Aurea Ruby Santiago‐Maury**: investigation, supervision, writing – original draft. **Sophia Sánchez López**: writing – original draft, investigation, visualization. **Iván de Jesús Leonor‐Hernández**: conceptualization, investigation, methodology. **Hector Cabello‐Rangel**: investigation, methodology, validation, visualization, writing – review and editing, supervision, formal analysis.

## Funding

The authors have nothing to report.

## Ethics Statement

This study was conducted in accordance with the Code of Ethics of the World Medical Association for Experiments Involving Human Subjects (Declaration of Helsinki). According to Mexican legislation, the study was classified as higher than minimal risk. In accordance with the protocol for Electroconvulsive Therapy of the Fray Bernardino Álvarez Psychiatric Hospital, informed consent was requested from the patient and responsible family member, and the patient and family member were also asked to sign an informed consent form to participate in the study. The study was approved by the hospital's Research and Research Ethics Committees (registration CI‐962).

## Conflicts of Interest

The authors declare no conflicts of interest.

## Data Availability

The authors declare the availability of data.
